# Health literacy across personality traits among older adults: cross-sectional evidence from Switzerland

**DOI:** 10.1007/s10433-023-00774-x

**Published:** 2023-06-28

**Authors:** Valérie-Anne Ryser, Clément Meier, Sarah Vilpert, Jürgen Maurer

**Affiliations:** 1 Swiss Centre of Expertise in the Social Sciences (FORS), c/o University of Lausanne, Lausanne, Switzerland; 2 Swiss Centre of Expertise in the Social Sciences (FORS), Faculty of Biology and Medicine (FBM) & The Faculty of Business and Economics (HEC), University of Lausanne, Lausanne, Switzerland; 3 Swiss Centre of Expertise in the Social Sciences (FORS), Faculty of Business and Economics (HEC), University of Lausanne, Lausanne, Switzerland; 4 Faculty of Business and Economics (HEC), Lausanne Center for Health Economics, Behavior and Policy (LCHE), Interdisciplinary Centre of Life Course Research (LIVES-UNIL), University of Lausanne, Lausanne, Switzerland

**Keywords:** Big five ten, Health literacy, Population-based study, SHARE, Switzerland

## Abstract

This research aims to better understand the association of personality traits (PT)—Openness to Experience, Conscientiousness, Extraversion, Agreeableness, and Neuroticism—with health literacy (HL) skills of adults aged 58 years and older in a nationally representative sample from Switzerland. Analyses were conducted on a subsample (n = 1546) of respondents living in Switzerland from wave 8 (2019/2020) of the Survey of Health, Ageing, and Retirement in Europe (SHARE). PT were assessed with the Big-Five inventory ten (BFI-10). HL was measured using the short version of the European Health Literacy Survey questionnaire (HLS-EU-Q16). We used multivariable regressions to explore how respondents' PT are independently associated with (1) the HLS-EU-Q16 and (2) seven sub-indices derived from this HL scale. Results demonstrated that even when controlling for social, regional, and health characteristics, PT were significantly associated with HL among older adults in Switzerland. More open individuals showed better HL competencies. By contrast, individuals who scored higher on neuroticism expressed more difficulties regarding concrete health-relevant tasks or situations. These findings call for public health policies targeting older adults with lower levels of openness who are less likely to engage in self-examination, and individuals with higher levels of neuroticism who tend to experience more negative emotions. Moreover, health information and communication strategies content development that accounts for different personality types and addresses the needs of individuals with low levels of openness and high neuroticism may help improve HL among older adults whose personalities may otherwise put them at a disadvantage in handling health information.

## Introduction

Individuals’ ability to find, understand, appraise, and use health information to handle health-related issues for themselves and others is reflected by their health literacy skills (Santana et al. 2021). Health literacy is, therefore, viewed as an important factor in ensuring patient autonomy, improving patient satisfaction, and achieving better outcomes in terms of health and healthcare (Kickbusch et al. 2013; Nielsen-Bohlman et al. 2004; Okan et al. 2019). Albeit health literacy is important throughout individuals’ life course, health literacy is particularly central at an older age. Aging populations are characterized by the increasing importance of physical and cognitive decline and rising rates of chronic disease. In such context, health literacy skills are particularly important since they influence how older adults recognize their health issues, communicate with healthcare providers, and make medical decisions (Ladin et al. 2018). Moreover, health literacy is also a strong predictor of health disparities between individuals and is associated with sociodemographic factors such as age, language, education, and social status (Levin-Zamir et al. 2016; Meier et al. 2022; Schaeffer et al. 2017; Sørensen et al. 2015). Health literacy skills are relevant for individuals’ health behavior, and despite the crucial role of health literacy in maintaining a good quality of life during the life course, little is known regarding interindividual differences in health literacy levels related to personality. Previous research mostly highlighted the importance of social factors and health status for health literacy (Kickbusch et al. 2013); however, little research has explored the role of personality and its association with health literacy (Iwasa and Yoshida 2020). Based on the Swiss Survey of Health Ageing and Retirement in Europe (SHARE) dataset, this study aims to fill this gap.

The Big Five personality construct is a well-established frame of reference in psychology (e.g. John and Srivastava 1999; McCrae and Costa 2003; McCrae and John 1992). It assesses how individuals position themselves relative to a list of statements. It aims to better understand individual differences in personality along five principal dimensions: Openness to experience, Conscientiousness, Extraversion, Agreeableness, and Neuroticism (John et al. 2008). Individuals with higher openness to experience tend to be more creative and open to new propositions or new intellectual experiences. Individuals with higher levels of conscientiousness tend to be more meticulous, responsible, hardworking, and more goal oriented. More extraversion is related to sociable, outgoing, and talkative individuals. Then, individuals with higher levels of agreeableness are usually more compliant and altruistic; they tend to act in a cooperative and unselfish manner. Finally, individuals with neurotic tendencies experience a chronic level of emotional instability and proneness to psychological distress; they may more frequently suffer from negative emotions such as anger, worry, and sadness. Personality traits thus reflect constant patterns of differences in how individuals think, feel, and behave (Roberts et al. 2007). In the context of healthy aging, personality traits play some crucial roles: they are associated with older adults’ healthcare utilization (Friedman et al. 2013; Hengartner et al. 2016; Nolan et al. 2019); positive health behaviors (Marks and Lutgendorf 1999), preventive healthcare use (Aarabi et al. 2021), negative health behaviors and outcomes such as alcohol consumption (Hakulinen et al. 2015a, b), smoking behavior (Hakulinen et al. 2015a, b), risk of diabetes (Jokela et al. 2014), functional impairments (Chapman et al. 2012; Hajek and König 2021; Krueger et al. 2006) and risk of death (Jokela et al. 2013). Recent evidence also showed that personality traits were associated with precautionary behavior against COVID-19 (Airaksinen et al. 2021). As personality traits shape individuals’ feelings, thinking, and behaviors and influence how individuals adjust to health-related issues, personality traits’ associations with individuals’ health literacy levels need to be further explored to promote health in older adults. A better understanding of the role of personality traits allows healthcare providers to tailor their communication with patients.

Based on the research mentioned above, personality dimensions are theoretically relevant to understanding better health literacy levels. Interindividual personality differences might directly or indirectly influence individuals’ health literacy because personality is related to broad temperamental tendencies. These general temperamental tendencies determine individuals’ cognitions, affects, and experiences. Therefore, each individual will interpret health-related issues and their surroundings differently. Personality traits will also influence how individuals deal with their health challenges. Moreover, personality traits will impact the way individuals experience different exposure to health-related outcomes. More concretely, from a theoretical perspective, the role of personality traits in shaping the ways individuals gain access to age and context-specific information from various sources seems to be obvious. In that sense, the different personality traits might be related to the way individuals are able to discriminate between multiple sources of information; personality traits influence how individuals understand and personalize health information that has been obtained; and finally, interindividual personality differences influence an individual’s ability to apply relevant health information for personal benefit appropriately.

## Research questions and hypotheses

The objective of this study is to shed light on the role of personality traits and their associations with health literacy. However, conceptually, health literacy is composed of several subdimensions and components. Therefore, one important further aim of our research is to better understand the association of personality traits not only with general health literacy levels but also with seven key subdimensions of health literacy: 1. Healthcare; 2. Disease prevention; 3. Health promotion; 4. Accessing/obtaining health information; 5. Understanding health information; 6. Processing/appraising health information; and 7. Applying/using health information.

To this end, we explore the following hypotheses: Since openness is linked with motivation to seek new experiences and to engage in self-examination (e.g. John and Srivastava 1999), we expect this personality trait to be related to the ease of finding health-related information. By contrast, lower levels of openness should be linked to feeling more comfortable with familiar and traditional experiences (e.g. John and Srivastava 1999). Besides conscientiousness is generally a major indicator of health, well-being, and longevity (e.g. John and Srivastava 1999), this trait is a determinant of maintaining long-term goals. Therefore, we argue that it might help individuals to address long-term consequences of the increasing frailty in aging or undergo long-term treatment in case of illnesses. Extraversion is, among other dimensions, linked with self-confidence (e.g. John and Srivastava 1999) and may thus contribute to processing and or appraising health-related issues and information with calm. Individuals who score higher on agreeableness tend to express more prosocial orientation, which likely allows them to have a large social network. Thus, these individuals may be exposed to a wide variety of sources of health information that could challenge their ability to discriminate between various sources of information. Finally, individuals with a high degree of neuroticism may express poorer reactions to illnesses and negative emotions. Such individuals may address health-related issues with negative emotions such as anxiety, anger, guilt, and depression, leading them to express poorer health literacy (e.g. John et al. 2008).

### Objectives of the present study

Given the potential separate benefits of health literacy and certain personality traits on health, studying the relationship between personality and health literacy can help highlight key opportunities and challenges for policies and interventions to promote healthy aging. Exploring the correlation between health literacy and personality traits among an aging population helps to better understand how older individuals’ attitudes resulting from their personality may be associated with health literacy skills and, thus, health behaviors. Despite the available literature on the benefits of health literacy (Kickbusch et al. 2013; Nielsen-Bohlman et al. 2004; Okan et al. 2019) and the association of personality traits with a large array of health behaviors (Aarabi et al. 2021; Chapman et al. 2012; Hajek and König 2021; Hakulinen et al. 2015a, b; Hakulinen et al. 2015a, b; Jokela et al. 2013; Krueger et al. 2006; Marks and Lutgendorf 1999), there are, as far as we know, no existing national representative studies to date that comprehensively describe the direct relationship between health literacy and personality traits among older adults. To the best of our knowledge, only one study based on a sample of community-dwelling older Japanese individuals investigates the associations between health literacy and its correlates, notably personality (Iwasa and Yoshida 2020). This lack of research on this field and the lack of representative studies based on random samples of older individuals lead us to address this gap in the literature. The aim of our research is to explore the correlations between health literacy and individuals’ personality traits in the Swiss-SHARE dataset. Our study presents several strengths: first, the question under interest is studied on a nationally representative sample of adults aged 58 years and older. The second strength is to consider a population that is not limited to community-dwelling individuals but older individuals in various kinds of accommodations. Finally, our study includes people at different stages of the aging process, i.e., people at the end of their professional career, young old, and the oldest old (Baltes and Smith 2003). These characteristics of our study provide greater scope for our results.

## Methods

### Study design and sample

Our data come from SHARE, which collects information on the health, socioeconomic status, and social networks of individuals aged 50 years and older using Computer-Assisted Personal Interviewing (CAPI). SHARE is a biennial population-based longitudinal study that started in 2004 and now includes 27 European countries and Israel (Börsch-Supan et al. 2013). SHARE samples are designed to be nationally representative of the target population of adults 50 years and older; thus, they are periodically refreshed to remain representative. Each survey round contains an internationally harmonized in-person interview and an optional country-specific paper-and-pencil self-administrated questionnaire. Our analyses are based on SHARE wave 8 data collected between October 2019 and the beginning of March 2020. They include respondents from Switzerland who participated in both the main questionnaire and the national paper-and-pencil self-administered questionnaire, which contained our health literacy outcome measure. On two occasions, respondents consented to participate in our study, first when they agreed to schedule an interview and second when they attended the in-person interview. We only include respondents aged 58 and older since the last refreshment sample for SHARE Switzerland was in 2011. The 8^th^ wave of SHARE included 2005 targeted respondents or their partners which means that the individuals’ response rate is 81%; among them, 94.3% also completed the national paper-and-pencil self-administrated questionnaire (N = 1891). Excluding respondents younger than 58 years old (partners of target respondents) and those with missing responses on variables used in the analysis, our final analytical sample counts 1546 respondents.

### Measures

#### Outcome variables

*Health literacy* Health literacy is assessed by the short version of the European Health Literacy Survey—HLS-EU-Q16 (Pelikan et al. 2019) developed by the HLS-EU consortium. This scale consists of 16 items of concrete health-relevant tasks or situations that respondents have to evaluate on a 4-point Likert scale with possible answers ranging from “very easy,” “fairly easy,” “fairly difficult,” to “very difficult” (Appendix 1). Each item is dichotomized (Pelikan et al. 2019) with a value of “0” if respondents expressed any difficulty (“fairly difficult” or “very difficult”) and “1” otherwise. Respondents with a maximum of one or two missing values on the 16 items are included in the analysis, with the missing values treated as a “0”. Adding all binary variables allows the construction of an aggregate health literacy score ranging from 0 to 16, which can be divided into three categories, i.e., inadequate (0–8), problematic (9–12), and sufficient health literacy (13–16). The internal consistency for the whole health literacy scale is excellent as shown by the Cronbach alpha (α = 0.91). Moreover, health literacy is composed of a large set of dimensions. To this end, the scale provides seven sub-indices measuring health literacy within three domains, i.e., healthcare (α = 0.83), disease prevention (α = 0.75), and health promotion (α = 0.79), as well as four stages of information processing, i.e., accessing/obtaining health information (α = 0.72), understanding health information (α = 0.78), processing/appraising health information (α = 0.67) and applying/using health information (α = 0.65). All the sub-indices present good to excellent internal consistency as shown by the Cronbach’s alphas ranging from 0.65 to 0.90. As the sub-indices contain a different number of items, they are standardized on a scale from a minimum of 0 to a maximum of 50, following the formula: Index = (mean − 1) × 50/3 (Sørensen et al. 2015).

#### Explanatory variable

*Personality* Personality traits were assessed using the 10-item version of the Big Five Inventory (BFI-10; Rammstedt and John 2007), an abbreviated version of the Big Five Inventory (John et al. 1991) developed by Rammstedt and John (2007). The BFI-10 is available in the general SHARE questionnaire. This extra short personality measure is specifically designed for large multi-topic panel surveys with great time constraints. Despite its brevity, the instrument has shown acceptable psychometric properties, meaning that it is a good compromise to measure personality. The scale contains two items for each of the five factors: Openness to Experience, Conscientiousness, Extraversion, Agreeableness, and Neuroticism. Respondents evaluate statements regarding their personality on a 5-point scale with possible answers: “strongly disagree”, “disagree”, “neither agree”, “not disagree”, “agree”, and “strongly agree” (Appendix 2). Each personality trait is thus evaluated on a scale from 1 to 5.

#### Independent and controlled variables

We adjusted the analysis for gender (0 = male, 1 = female), age group (58–64 years, 65–74 years, 75+years), and education level (low = 0–2, secondary = 3–4, tertiary = 5–6) using the “International Standard Classification of Education” (Hoffmeyer-Zlotnik and Wolf 2003). We also controlled for the presence of a partner in the household (0 = has a partner, 1 = has no partner), the self-perceived financial situation as measured by the question: "Is your household able to make ends meet?" with answers being coded as 1 = “easily”, 2 = “fairly easily”, and 3 = “with difficulty”. The language used to answer the questionnaire helps to differentiate Switzerland’s three main linguistic regions (Swiss-German, French, or Swiss-Italian). Further information on the living environment comes from a variable assessing whether respondents lived in an urban or rural area (0 = urban, 1 = rural). Finally, we added two variables assessing respondents’ health status: first, a measure of self-rated health (1 = “poor/fair”, 2 = “good”, 3 = “very good/excellent”); second a measure of the respondent’s difficulties with a range of activities of daily living (ADL) evaluating if respondents had any limitations (0 = no, 1 = yes) with a list of activities (ADL; Katz et al. 1963; Steel et al. 2003).

### Statistical methods

As advised, cross-sectional weights provided in the SHARE data (Börsch-Supan 2022) have been used to calibrate the sample to obtain descriptive statistics representative of the population of interest. We also determined the distribution of health literacy scores per personality traits level using weighted proportion. Partial associations between health literacy levels with respondents’ personality traits and sociodemographic characteristics were estimated using unweighted ordinary least squares (OLS) regression (health literacy score) and multivariable ordered probit model (health literacy grouped), reporting results in terms of average marginal effects (AME). By testing these associations on two different statistical models, we can examine the robustness of our estimates. In addition, we used unweighted OLS regression to determine the partial association between the standardized health literacy score and its seven sub-indices with respondents’ personality traits and sociodemographic characteristics. Finally, as the SHARE study surveyed the targeted respondents and the partner, we accounted for the possibility of dependencies in the observations by clustering the estimated standard errors at the household level. The analyses were performed using STATA/SE 17.0 (STATA Corporation, College Station, TX) with statistical significance levels of *p < 0.05, **p < 0.01, ***p < 0.001.

## Results

Table 1 displays the numbers for both the unweighted and the weighted sample. Our weighted analytical sample contained 48.2% of women (Table 1); the average age was 71.6 years old (SD: 8.4), with 26.9% of respondents aged between 65 and 75 years old and 23.3% older than 75 years old. Regarding the education level, 16.1% had a low level of education, 63.4% had a secondary level education, and the remaining respondents had at least a tertiary education. Most respondents had a partner (70.5%), no financial difficulties (87.1%), came from the German-speaking part of Switzerland (71.0%) and lived in a rural area (58.3%). Finally, most respondents self-rated their health as good or excellent (83.1%), and 6.5% had one or more difficulties with ADL.

**Table 1:** Characteristics of the study population, adults aged 58+, SHARE Switzerland, 2019/2020, n = 1546

	Unweighted	Weighted	
	N	%	95% CI
*Gender*			
Male	726	51.8	[47.4–56.1]
Female	820	48.2	[43.9–52.6]
*Age groups*			
58–64 years	375	49.8	[44.7–54.9]
65–74 years	635	26.9	[23.8–30.2]
75+ years	536	23.3	[20.6–26.3]
*Education*			
Low	279	16.1	[13.2–19.6]
Secondary	968	63.4	[58.7–67.8]
Tertiary	299	20.5	[16.7–24.9]
*Partnership status*			
Has a partner	1151	70.5	[65.9–74.7]
No partner	395	29.5	[25.3–34.1]
*Make ends meet*			
Easily	856	56.9	[52.2–61.6]
Fairly easily	490	30.2	[26.2–34.6]
With difficulty	200	12.8	[10.0–16.3]
*Language*			
German	1106	71.0	[66.1–75.4]
French	385	26.1	[21.8–31.0]
Italian	55	2.9	[1.9–4.3]
*Living area*			
Urban	709	41.7	[37.0–46.6]
Rural	837	58.3	[53.4–63.0]
*Self-rated health*			
Poor/fair health	297	16.9	[13.8–20.4]
Good health	660	38.9	[34.5–43.6]
Very good/excellent health	589	44.2	[39.2–49.4]
*ADL limitations*			
No	1437	93.5	[91.1–95.3]
Yes	109	6.5	[4.7–8.9]

Unweighted and weighted number of observations for the whole sample. N = number; CI = confidence interval

**Fig. 1:**
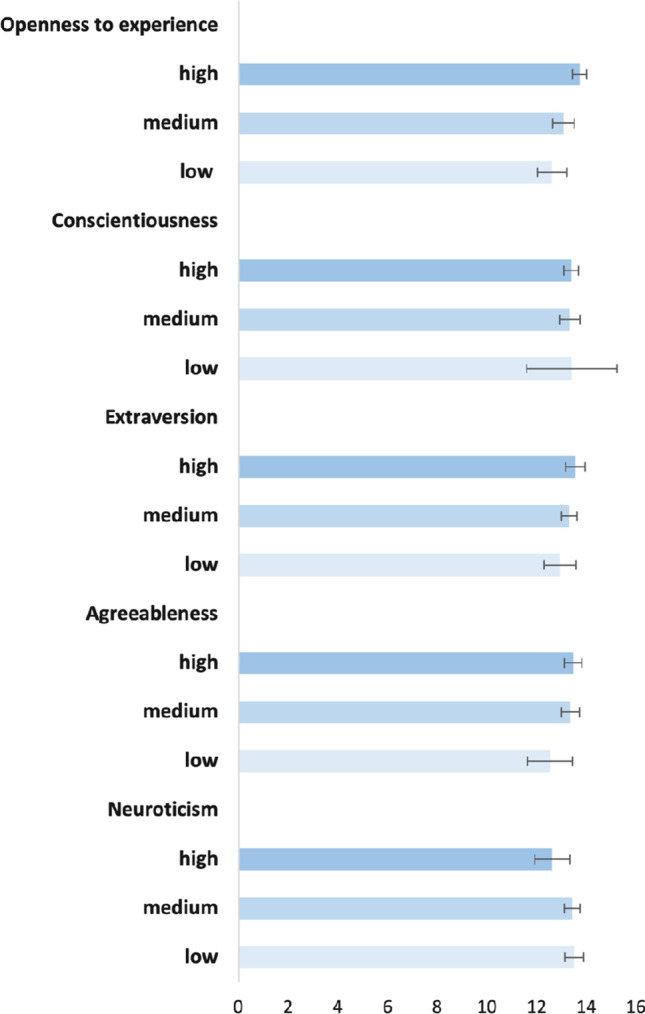
Health literacy’s weighted average scores per personality traits levels and 95% confidence intervals, adults aged 58+, SHARE Switzerland, 2019/2020, n = 1546. The health literacy score ranges from 0 to 16, while the personality traits levels are regrouped into three categories: low (1; 1.5; 2), medium (2.5; 3; 3.5) and high (4; 4.5; 5)

The weighted distribution of health literacy scores for each personality trait level showed significant associations for three of the five traits (Fig. 1). Individuals expressing being more open to experiences (*p* < 0.001) and those with higher levels of extraversion seemed to have higher health literacy levels (*p* = 0.026), while respondents with higher scores on neuroticism tended to have lower health literacy scores (*p* = 0.031).

Table 2 presents the partial associations of health literacy with respondents’ personality traits and sociodemographic characteristics. Regarding personality traits, individuals with a higher degree of openness to experiences were more likely to have higher health literacy scores (*p* < 0.001) and were less likely to have problematic and inadequate levels of health literacy (*p* < 0.001). Respondents expressing higher neuroticism had lower health literacy scores (*p* < 0.01) and were more likely to have problematic and inadequate levels of health literacy (*p* < 0.05). Concerning the associations with individuals’ social, regional, and health characteristics, multivariable analyses indicated that—holding other characteristics fixed—health literacy was lower in men, individuals with low levels of education, individuals with trouble making ends meet, and those with poor self-rated health or ADL limitations.

**Table 2:** Partial associations of health literacy with respondents’ personality traits and sociodemographic characteristics, adults aged 58+, SHARE Switzerland, 2019/2020, n = 1546

	OLS regression (HL score)	Oprobit (problematic) AME (SE)	Oprobit (inadequate) AME (SE)
Gender (male)			
Female	0.64***	− 0.03***	− 0.06***
	(0.14)	(0.01)	(0.01)
Age group (58–64 years)			
65–74 years	0.12	− 0.01	− 0.01
	(0.19)	(0.01)	(0.02)
75+years	− 0.08	0.01	0.02
	(0.21)	(0.01)	(0.02)
Education (low)			
Secondary	0.33	− 0.02	− 0.03
	(0.22)	(0.01)	(0.02)
Tertiary	1.03***	− 0.05***	− 0.11***
	(0.24)	(0.01)	(0.03)
Partnership status (has a partner)			
No partner	0.19	− 0.01	− 0.02
	(0.18)	(0.01)	(0.02)
Make ends meet (easily)			
Fairly easily	− 0.19	0.01	0.02
	(0.17)	(0.01)	(0.02)
With difficulty	− 0.85**	0.04*	0.06*
	(0.27)	(0.02)	(0.02)
Language (Swiss-German (ch))			
French (ch)	0.09	0.00	0.01
	(0.19)	(0.01)	(0.02)
Swiss-Italian (ch)	− 0.34	0.01	0.02
	(0.48)	(0.03)	(0.05)
Living area (urban)			
Rural	− 0.12	0.01	0.02
	(0.15)	(0.01)	(0.02)
Self-rated health (bad health)			
Good health	0.59**	− 0.02	− 0.04
	(0.23)	(0.01)	(0.02)
Very good/excellent health	1.13***	− 0.06***	− 0.10***
	(0.23)	(0.01)	(0.02)
ADL limitations (no)			
Yes	− 1.83***	0.12***	0.13***
	(0.39)	(0.03)	(0.02)
Openness (Big Five personality inventory)	0.29***	− 0.02***	− 0.03***
	(0.08)	(0.00)	(0.01)
Conscientiousness (Big Five personality inventory)	0.01	0.00	0.00
	(0.11)	(0.01)	(0.01)
Extraversion (Big Five personality inventory)	0.08	− 0.00	− 0.01
	(0.08)	(0.00)	(0.01)
Agreeableness (Big Five personality inventory)	− 0.15	0.01	0.01
	(0.10)	(0.01)	(0.01)
Neuroticism (Big Five personality inventory)	− 0.23**	0.01*	0.02*
	(0.08)	(0.00)	(0.01)
Observations	1546	1546	1546

Ordinary Least Squares (OLS) regression of the Health Literacy (HL) score on the covariates, and an oprobit regression of the three-category HL score on the covariates. The table shows average marginal effects (AMEs) and standard errors in brackets with significance level: *p < .05, **p < .01, ***p < .001

The second part of the analysis considers the link between personality traits and the sub-dimensions of the health literacy scale for whom hypotheses have been developed. The associations between the standardized health literacy score and the seven standardized sub-indices with respondents’ personality traits and sociodemographic characteristics yielded similar results regarding gender, education, self-rated health, ADL limitations, openness to experience, and neuroticism (Table 3). However, we also found some statistically significant associations with other personality traits. Respondents with a lower level of conscientiousness were more likely to report greater ease in understanding health information (*p* < 0.05). Having a higher level of extraversion was associated with higher health literacy scores (*p* < 0.05), higher healthcare-related health literacy (*p* < 0.01), facilities in obtaining health-related information (*p* < 0.001), and greater ease in applying health information (*p* < 0.05). Finally, respondents with higher levels of agreeableness had lower healthcare-related health literacy (*p* < 0.05) and were more likely to report difficulties appraising health information (*p* < 0.05).

**Table 3:** Partial associations of health literacy sub-indices with respondents’ personality traits and sociodemographic characteristics, adults aged 58+, SHARE Switzerland, 2019/2020, n = 1546

	HL score	Healthcare (hc_hl)	Disease prevention (dp_hl)	Health promotion (hp_hl)	Access/obtain (oi)	Under-standing (ui)	Process/appraise(pi)	Apply/use (ai)
Gender (male)								
Female	1.77***	1.54***	1.76***	2.19***	2.03***	1.57***	2.17***	1.44**
	(0.36)	(0.39)	(0.43)	(0.46)	(0.43)	(0.38)	(0.50)	(0.44)
Age group (58–64 years)								
65–74 years	0.25	0.43	0.41	− 0.25	0.10	0.49	− 0.33	− 0.10
	(0.49)	(0.50)	(0.58)	(0.61)	(0.58)	(0.49)	(0.66)	(0.58)
75+years	− 0.06	0.98	− 0.72	− 1.05	− 0.56	− 0.02	− 0.03	0.52
	(0.54)	(0.55)	(0.65)	(0.67)	(0.65)	(0.55)	(0.71)	(0.63)
Education (low)								
Secondary	1.09*	1.00	1.14	1.16	0.90	1.44**	0.83	0.88
	(0.53)	(0.56)	(0.64)	(0.70)	(0.63)	(0.56)	(0.71)	(0.64)
Tertiary	3.57***	3.35***	3.34***	4.23***	3.15***	4.38***	2.92**	3.16***
	(0.65)	(0.66)	(0.79)	(0.88)	(0.78)	(0.67)	(0.89)	(0.80)
Partnership status (has a partner)								
No partner	1.02*	0.87	1.10*	1.17*	0.72	1.27**	1.03	0.88
	(0.46)	(0.48)	(0.55)	(0.58)	(0.56)	(0.47)	(0.61)	(0.57)
Make ends meet (easily)								
Fairly easily	− 0.92*	− 0.98*	− 1.20*	− 0.48	− 1.19*	− 0.90*	− 0.95	− 0.58
	(0.43)	(0.45)	(0.52)	(0.55)	(0.52)	(0.44)	(0.58)	(0.52)
With difficulty	− 2.27***	− 2.78***	− 1.71*	− 2.08*	− 3.82***	− 2.32***	− 1.06	− 1.33
	(0.65)	(0.70)	(0.76)	(0.82)	(0.81)	(0.65)	(0.84)	(0.79)
Language (German (ch))								
French (ch)	− 0.68	− 1.02*	0.07	− 1.00	− 1.06	− 1.40**	1.01	− 0.41
	(0.48)	(0.50)	(0.57)	(0.60)	(0.58)	(0.47)	(0.62)	(0.58)
Italian (ch)	− 1.73	− 0.93	− 2.64*	− 2.00	− 2.26	− 2.77*	0.48	− 1.16
	(1.19)	(1.20)	(1.32)	(1.46)	(1.44)	(1.36)	(1.40)	(1.19)
Living area (urban)								
Rural	− 0.28	− 0.15	− 0.24	− 0.55	− 0.08	− 0.55	0.08	− 0.36
	(0.39)	(0.40)	(0.46)	(0.48)	(0.47)	(0.39)	(0.51)	(0.47)
Self-rated health (poor/fair health)								
Good health	1.13*	0.87	1.07	1.64*	1.45*	0.89	1.29	0.99
	(0.53)	(0.58)	(0.63)	(0.67)	(0.66)	(0.55)	(0.71)	(0.64)
Very good/excellent health	2.86***	2.29***	2.64***	4.13***	3.06***	2.54***	3.89***	2.20**
	(0.58)	(0.62)	(0.68)	(0.73)	(0.72)	(0.59)	(0.78)	(0.70)
ADL limitations (no)								
Yes	− 3.48***	− 4.23***	− 2.48**	− 3.42**	− 4.40***	− 3.00***	− 2.85**	− 3.84***
	(0.83)	(0.95)	(0.96)	(1.11)	(0.97)	(0.91)	(1.05)	(1.10)
Openness	1.01***	0.81***	1.09***	1.24***	1.14***	1.04***	1.02***	0.74**
	(0.22)	(0.22)	(0.26)	(0.28)	(0.26)	(0.22)	(0.30)	(0.26)
Conscientiousness	0.48	0.46	0.51	0.47	0.33	0.57*	0.48	0.48
	(0.27)	(0.27)	(0.32)	(0.37)	(0.32)	(0.27)	(0.37)	(0.32)
Extraversion	0.44*	0.67**	0.32	0.17	0.82***	0.08	0.49	0.57*
	(0.21)	(0.22)	(0.24)	(0.27)	(0.25)	(0.22)	(0.28)	(0.25)
Agreeableness	− 0.47	− 0.54*	− 0.43	− 0.41	− 0.44	− 0.39	− 0.72*	− 0.44
	(0.25)	(0.27)	(0.30)	(0.33)	(0.32)	(0.25)	(0.35)	(0.30)
Neuroticism	− 0.70***	− 0.75***	− 0.66**	− 0.67**	− 0.87***	− 0.51*	− 0.94***	− 0.61*
	(0.20)	(0.21)	(0.24)	(0.26)	(0.25)	(0.21)	(0.26)	(0.25)
Constant	29.23***	31.41***	27.09***	28.09***	28.55***	32.18***	24.58***	28.88***
	(1.93)	(2.06)	(2.20)	(2.51)	(2.29)	(2.00)	(2.52)	(2.23)
Observations	1546	1546	1546	1546	1546	1546	1546	1546

Regressions of the standardized health literacy score and the sub-Indices on covariates. Sub-indices abbreviations: health care (hc_hl), disease prevention (dp_hl), health promotion (hp_hl), access/obtain health information (oi), understanding health information (ui), process/appraise health information (pi), apply/use health information (ai). Estimates and standard errors in parentheses, significance level: *p < .05, **p < .01, ***p < .001

## Discussion

To the best of our knowledge, our study is the first representative population-based study exploring the associations between different personality traits and the level of health literacy among older adults. When considering the entire health literacy scale, we found that individuals expressing being more open to experiences were more likely to have higher health literacy levels. This result may reflect that more open individuals tend to seek new experiences that might help them to access specific information from various sources, to seek appropriate healthcare services, and to find health-related information (e.g. John et al. 2008; John and Srivastava 1999), which are crucial components of effective health literacy. More open individuals also have a more fluid style of awareness (e.g. John et al. 2008) that allows them to make new associations between distantly related ideas that might help them adopt a more comprehensive understanding of their health status. In addition, more open individuals tend to engage in self-examination (e.g. John and Srivastava 1999), which may make them more aware of their health situation and help them better appraise and use health-related information.

Second, individuals with higher scores on neuroticism were more likely to have inadequate health literacy. Individuals with higher scores on neuroticism are also more prone to negative emotions, express lower emotional intelligence, and are at risk for mental disorders such as phobia, depression, panic fears, and other anxiety disorders (e.g. John et al. 2008), which may involve decreased motivation and interpersonal activities. These characteristics might slow down or diminish individuals’ ability to seek, understand and appraise health-related information, as well as negatively influence their judgment and decision. Finally, neuroticism is a function of the limbic system activity (Eysenck 1983) which means that individuals with a higher neuroticism score have a reactive sympathetic nervous system that is more sensitive to the environment around them. Individuals with higher neuroticism might overreact and become very anxious when facing health-related information from different healthcare providers; in addition, facing health related-outcomes such as the symptoms of an illness can also generate extra negative emotions. Therefore, higher levels of neuroticism may undermine their health literacy skills.

One strength of our study is that we adopt a rather detailed perspective by also considering the multiple sub-dimensions of health literacy linked with respondents’ personality traits. Some of our hypotheses align with the results: respondents with higher levels of conscientiousness were more likely to report greater ease in understanding health information. This finding may reflect that conscientious individuals tend to be more organized and more goal-oriented (e.g. John and Srivastava 1999), which helps them to better understand health-related issues and behave accordingly. Respondents with higher levels of agreeableness were more likely to report difficulties regarding healthcare-related health literacy and to report difficulties appraising health information. Individuals with higher levels of agreeableness are generally more trusting, tolerant, compliant, and cooperative (e.g. John and Srivastava 1999). For those individuals, difficulties appraising health information might be generated by their social network, which may transmit conflicting health information: being more trusting (e.g. John and Srivastava 1999) may reduce individuals’ capacity to appropriately appraise and select health information from individuals surrounding them. Finally, individuals with higher levels of extraversion have higher health literacy scores, especially in the healthcare domain, which might be linked with self-confidence. Higher competence in obtaining health information may stem from being more talkative and having higher social capacity. Their greater ease in applying health information may originate from higher levels of self-confidence of these individuals (e.g. John and Srivastava 1999).

### Limitations

Despite its numerous strengths, our study suffers from several limitations. Firstly, HLS-EU-Q16 (Pelikan et al. 2019) and the Big Five Inventory Ten (BFI-10; Rammstedt and John 2007) are subjective measures that may be subject to reporting heterogeneity and bias in case of systematic reporting differences. Regarding the health literacy scale, respondents may over or underestimate their skills. However, subjective health literacy assessments seem to be the most appropriate method for self-administered paper-and-pencil questionnaires, as when using test-based evaluations, it is not possible to prevent respondents from consulting the internet or asking household members to help them answer the questions, which could significantly bias the results from a more test-based approach to assessing health literacy. In addition, the short version of the HLS-EU-Q16 questionnaire has several advantages, including convenience and validity (Eronen et al. 2019; Lorini et al. 2019). Indeed, health literacy represents individuals' rating of their perception of their own health-related skills. In that sense, health literacy is linked with self-efficacy and control beliefs of individuals' healthcare-related tasks (Berens et al. 2022). Future research should, however, better target other aspects of the health literacy construct: more objective components of health literacy should be studied to have a more comprehensive and exhaustive understanding of the complexity of this construct.

Regarding the BFI-10 (Rammstedt and John 2007), despite its brevity, the instrument has shown acceptable psychometric properties (Steyn and Ndofirepi 2022). High reliability and validity when measuring individuals' personality traits have been demonstrated (Rammstedt and John 2007). That is, albeit ultra-short personality measures present the advantage of avoiding items redundancy and allowing the measurement of psychological construct in surveys when time is severely limited, they are, to some extent, inferior to regular personality assessment (Gosling et al. 2003; Rammstedt and John 2007). They should not substitute to them. A disadvantage of extra short personality trait measures is their inability to assess individual facets of multi-faceted constructs of personality traits (Gosling et al. 2003). In addition, psychological scales were originally constructed for personal assessment purposes with questionnaires administered to individuals by trained professionals. In large interdisciplinary surveys like SHARE, these scales must be adapted to assess psychological constructs in the whole population (Rammstedt and Beierlein 2014). Moreover, it is trained interviewers, but not trained psychologists or psychotherapist, who administer or read the surveys questions (Ryser 2023). Furthermore, recent research based on lexical studies of personality structure emphasized the importance of the Ashton and Lee (2014) HEXACO six-factor model. This model partially overlaps the big five model but highlights the importance to considers an additional Honesty-Humility factor in future research (Ashton et al. 2014). Finally, personality traits are relatively enduring patterns with biological bases (McCrae and Costa Jr 1996), and despite previous research supporting personality traits’ one-direction associations with health literacy (Iwasa and Yoshida 2020), reverse causality cannot be ruled out. The study design does not allow causality to be determined. However, reverse causality should be better explored and understood in future research, especially in light of recent developments in the research of the evolution of personality traits over the life span (e.g. Graham et al. 2020) and as a function of life events (Bleidorn et al. 2018).

Lastly, selection effects and attrition add concerns about the representativeness of SHARE samples. However, this problem is common in all longitudinal and population-based surveys (Banks et al. 2011). Moreover, considerable effort is being made to minimize these biases in the SHARE survey in Switzerland. Furthermore, missing responses did not appear to be a significant concern in our study as it was relatively low (18.2%). No population group appeared to be systematically over-represented among the respondents excluded from our analyses because of item non-response.

## Conclusion

Previous research has shown that one-third of Swiss older adults have difficulty managing their health (Meier et al. 2022). Low levels of health literacy negatively impact individuals’ quality of life, particularly in older adults with a more vulnerable health status (Ladin et al. 2018). In our study, we found that individuals with relatively low openness to experience and high neuroticism were particularly at risk of presenting inadequate health literacy levels. Public health policies should focus on individuals with lower openness levels, which is linked with a lower capacity to engage in self-examination, and those displaying higher neuroticism, which may be more anxious when dealing with health-related information. These policies should thus incorporate personality traits in the personalization of services, such as in health or eHealth communication tools for the general population and the training of healthcare providers. For instance, public health policies aiming at improving health literacy could be more convincing or nudge-driven for older adults with low openness as well as more factual and calming for those with high neuroticism.

## Data Availability

This article uses data from Börsch-Supan (2022). Survey of Health, Ageing and Retirement in Europe (SHARE) Wave 8. Release version: 8.0.0. SHARE-ERIC. Data set. https://doi.org/10.6103/SHARE.w8.100. Study data already deidentified are available to the scientific community upon submitting a data requestion application to the SHARE study.

## Appendices

**Table:**

Appendix 1: The 16 items from the HLS-EU-Q16 scale Pelikan, Ganahl, Van den Broucke, Sorensen (2019):
First, we would like to ask you how comfortable you feel when dealing with health-related information
For you, how easy or difficult is it to…
Answer categories: “Very easy”, “Fairly easy”, “Fairly difficult”, “Very difficult”
Healthcare
1. Understand your doctor’s or pharmacist’s instructions on how to take a prescribed medicine?
2. Follow instructions from your doctor or pharmacist?
3. Understand what doctor says to you?
4. Find out where to get professional help when you are ill?
5. Find information on treatments of illnesses that concern you?
6. Use the information the doctor gives you to make decisions about your illness?
7. Judge when you may need to get a second opinion from another doctor?
Disease prevention
8. Understand health warnings about behaviour such as smoking, low physical activity, and drinking too much?
9. Understand why you need health screenings?
10. Find information on how to manage mental health problems like stress or depression?
11. Decide how you can protect yourself from illness based on information in the media?
12. Judge if the information on health risks in the media is reliable?
Health promotion
13. Understand advice on health from family members or friends?
14. Judge which everyday behaviour is related to your health?
15. Find out about activities that are good for your mental well-being?
16. Understand information in the media on how to get healthier?

**Table:**

Appendix 2: The items from the Big Five personality inventory (BFI-ten Rammstedt and John 2007):	
Openness to experience	I see myself as someone who has few artistic interests
	I see myself as someone who has an active imagination
Conscientiousness	I see myself as someone who tends to be lazy
	I see myself as someone who does a thorough job
Extraversion	I see myself as someone who is reserved
	I see myself as someone who is outgoing, sociable
Agreeableness	I see myself as someone who is generally trusting
	I see myself as someone who tends to find fault with others
Neuroticism	I see myself as someone who is relaxed, handles stress well
	I see myself as someone who gets nervous easily
Answer categories: “Disagree strongly”, “Disagree a little”, “Neither agree nor disagree”, “Agree a little”, “Agree strongly”	
